# Catastrophe mechanism and early warning indicators of seepage erosion-induced water inrush in karst cavities

**DOI:** 10.1371/journal.pone.0354695

**Published:** 2026-07-31

**Authors:** Jiejun Yuan, Peng Zhang, Xiaqing Qian, Lingli Zhang, Yajing Liu

**Affiliations:** 1 Jiangsu Power Transmission and Transformation Co., Ltd., Nanjing, China; 2 School of Transportation Engineering, Nanjing Tech University, Nanjing, Jiangsu, China; 3 College of Civil Engineering, Jiangsu Open University, Jiangsu, Nanjing, China; China University of Mining and Technology, CHINA

## Abstract

Seepage-erosion-induced water inrush in karst cavities is a typical form of water-inrush disaster in karst tunnels. It is governed not only by the spatial distribution of karst cavities and hydraulic recharge conditions, but also by the particle-size gradation and composition of the cavity fill. During seepage erosion, fill particles are progressively transported by flowing water, which may trigger a sudden water-inrush catastrophe. To reveal the catastrophe mechanism and establish early-warning indicators, this study employs the Smoothed Particle Hydrodynamics (SPH) method to simulate the evolution of seepage-erosion-induced water inrush under different particle-size gradations, cavity confining stresses, and seepage velocities. The inflection point of the cumulative particle loss rate is used as an indicator of catastrophic transition. The relationships among particle-size gradation, confining stress, seepage velocity, and particle loss rate at the transition point are then analyzed to determine early-warning thresholds. The results show that fill-particle loss is positively correlated with both seepage velocity and confining stress. When the content of fine particles, such as rock cuttings and fine sand, exceeds 60%, the inflection point corresponds to a seepage velocity of 1.6 m/s and a confining stress of 2.6 MPa, with an early-warning particle-loss range of 8%−15%. When the content of coarse particles, such as coarse sand and gravel, exceeds 40%, the inflection point corresponds to a seepage velocity of 3.0 m/s and a confining stress of 4.5 MPa, with an early-warning particle-loss threshold of approximately 45%.

## 1. Introduction

Water inrush is a common geological hazard during the construction of mountain tunnels through water-bearing structures. The advance detection of water-bearing structures and the monitoring and early warning of water-inrush disasters are therefore critical for construction safety in water-rich strata. Once a water-inrush disaster occurs, it can seriously delay construction and increase project cost [1–3]. According to the type of karst water-bearing structure, tunnel water inrush can be classified as fissure-type, cavity-type, fault-type, and underground-river-type water inrush. The corresponding mechanical mechanisms mainly include hydraulic fracturing, slip failure, collapse and fragmentation, seepage erosion, and direct exposure [4,5]. Because conventional advance geological forecasting methods have limited ability to identify concealed karst cavities and their hydraulic connectivity, cavity-type water inrush remains one of the most difficult and hazardous forms of karst tunnel disaster to prevent.

Current research on water-inrush disasters in karst tunnels has mainly focused on advance geological prediction of water-bearing structures [6], disaster evolution and catastrophe mechanisms [7–9], prediction and early warning, and prevention and control measures [10]. Among these topics, the simulation of disaster evolution provides an important basis for understanding catastrophe mechanisms and identifying monitoring indicators. Physical model tests can directly re-produce the mechanical evolution and hydraulic response of water-inrush processes and have therefore been widely used in this field [11]. Li [12] developed a simulation system for water and sand inrush induced by mining-related overburden failure. Li et al. [13,14] developed a fluid-solid coupling model test system and systematically investigated water-inrush evolution under different geological conditions, such as faults and karst structures, by monitoring displacement, stress, and seepage pressure during rock failure. Chen et al. [15,16] conducted large-scale model tests on ground collapse caused by water and sand inrush in subway tunnels. Yang [17] and Liu [18] studied the relationship between porosity and permeability during water-sand inrush at the sample scale. Numerical simulation has also become an effective tool for observing internal disaster-evolution processes. Zhou [19] used a coupled DEM-CFD method to study seepage failure at the sample scale and catastrophic inrush at the engineering scale. Gao [20] and Ma et al. [21] used the discontinuous deformation analysis method to simulate water inrush in water-rich fault zones and high-pressure karst tunnels. Zhao Ning [22] used CFD, Darcy-PDE coupling, and COMSOL multi-physics models to analyze water-inrush patterns in clay-filled faults, fault fracture zones, and fractured rock masses. Zhou [23–25] described the flow behavior of materials in filled pipelines using the Navier-Stokes equations. Sun [26] applied the SPH method to simulate disaster evolution and water inrush in filled structures. Wang et al. [27,28] investigated non-Darcy flow and turbulent-flow evolution in fault fracture zones and karst pipelines using PDE-based methods.

Water-inrush disasters in karst cavities are generally controlled by three mechanisms: slip failure, seepage erosion, and direct exposure. Among them, seepage-erosion-induced water inrush is a complex failure mode involving both progressive erosion and sudden outburst [29,30]. Karst formations commonly contain interconnected cavities that may be partially filled with rock debris and sediments. When tunnel excavation exposes such cavities, strong groundwater seepage pressure and excavation disturbance may first induce small-scale water gushing. High-pressure water then seeps along cavity walls and through the cavity fill, carrying away clay and fine particles and gradually reducing the internal stability of the fill. This process may develop into water-mud or water-sand inrush and finally evolve into a full water-inrush disaster. Mechanically, the process represents a transition from stable seepage to unstable non-Darcy flow and involves coupled changes in strength, permeability, and apparent viscosity [31–34]. Existing physical and numerical studies of seepage-erosion-induced karst-cavity water inrush have mainly focused on water-inrush volume and the influence of fill-particle gradation. However, the evolution of mud or sand discharge and the influence of confining stress on the particle gradation and density of the cavity fill remain insufficiently addressed.

Numerical simulation is particularly useful for observing the internal evolution of water-inrush processes. However, seepage-erosion-induced water inrush in karst cavities involves both solid-fluid interaction and the coupled movement of fluid and granular particles. Neither a purely discrete-element model nor a purely fluid-dynamics model can fully describe this process. In addition, DEM-CFD coupling depends on semi-empirical drag-force formulations whose reliability may vary with particle gradation and flow regime [35–38]. In this study, the SPH method is used to simulate both water flow and cavity-fill particles within a unified particle framework. This enables the transition from stable seepage to unstable seepage-induced catastrophe to be modeled directly. The effects of fill composition and gradation, seepage velocity, fill density, and confining stress are systematically examined. The temporal relationship between water discharge and mud/sand discharge is analyzed, and the inflection point of the cumulative particle loss rate is used as a criterion for identifying catastrophic transition. Based on this criterion, the relationships among particle-size gradation, confining stress, seepage velocity, and particle loss rate are quantified, and early-warning indicators for seepage-erosion-induced karst-cavity water inrush are proposed.

## 2. SPH model for seepage erosion-induced karst cavity water inrush

### 2.1. Basic theory and method of SPH

SPH method is a Lagrangian meshless particle method that approximates physical problems using kernel functions. It describes macroscopically continuous and microscopically discrete fluids using discrete particles, with each particle carrying various properties of the fluid at its location, such as mass, density, velocity, and energy.

The basic principles are as follows: (1) When solving fluid dynamics problems using the SPH method, the problem domain is represented by a series of arbitrarily distributed particles that are not connected to each other and are initially arranged uniformly; (2) Field functions are approximated using the integral kernel method; (3) Particle approximation of field functions is achieved by summing the corresponding values of neighboring particles within the support domain; (4) The particle approximation method is applied to all field function-related terms in the system of partial differential equations, which are then discretized to solve the problem using a pure Lagrangian approach.

The SPH method involves a two-step solution process: first, the function is approximately expressed using an integral form, and then the function value at a discrete point is approximated by summing the values of the nearest neighboring particles. For detailed formulas of the SPH method, please refer to literature [39–43], which will not be repeated here.

### 2.2. Cavity fill materials

The fill materials in the cavity have a significant impact on seepage erosion-induced inrush water. This paper considers three types of fill materials: clayey, silty, and gravelly, with their particle size distribution and grading parameters shown in Tables 1-2, 3:

**Table 1:** Basic parameters of clayey fill.

Particle size range/mm	Particle content/%	Grading parameters	
**2-5**	0	Effective diameter d10/mm	0.075
**0.5-2**	3.6	Limiting diameter d30/mm	0.075
**0.25-0.5**	3.7	Limiting diameter d60/mm	0.075
**0.075-0.25**	15.6	Coefficient of uniformity Cu	1
**<0.075**	77.1	Coefficient of curvature Cc	1

**Table 2:** Basic parameter of clay-sand fill.

Particle size range/mm	Particle content/%	Grading parameters	
**2-5**	2.3	Effective diameter d10/mm	0.075
**0.5-2**	32.4	Limiting diameter d30/mm	0.2
**0.25-0.5**	28.8	Limiting diameter d60/mm	0.432
**0.075-0.25**	25.6	Coefficient of uniformity Cu	5.76
**<0.075**	10.9	Coefficient of curvature Cc	1.23

**Table 3:** Basic parameter of gravel fill.

Particle size range/mm	Particle content/%	Grading parameters	
**2-5**	22.8	Effective diameter d10/mm	0.394
**0.5-2**	60.7	Limiting diameter d30/mm	0.792
**0.25-0.5**	7.6	Limiting diameter d60/mm	1.486
**0.075-0.25**	5.8	Coefficient of uniformity Cu	3.77
**<0.075**	3.1	Coefficient of curvature Cc	1.07

(1) Clay type: Predominantly composed of silt and clay particles, with a small amount of fine sand particles, and very little or no coarse sand, gravel, or cobble content. The medium is densely packed.
(2) Clay-sand type: A balanced proportion of gravel, coarse sand, medium sand, fine sand, and silt/clay particles. The voids between coarse particles are filled by medium particles, and the voids between medium particles are filled by fine particles. The particles are mainly in concave contact, resulting in small pores between them.
(3) Gravel type: Predominantly composed of boulders, gravel, and coarse sand, with a supplementary amount of medium sand, and very little or no fine sand or silt/clay content. The particles are mainly in point contact, with large voids between coarse particles that cannot be reasonably filled, leading to high permeability.

Particle size range and mass fraction are the two key parameters controlling the particle-size gradation of the cavity fill. In this study, the particles were classified into five size ranges: 2–5 mm, 0.5–2 mm, 0.25–0.5 mm, 0.075–0.25 mm, and <0.075 mm. The filling material was prepared by mixing particles from these five ranges while keeping the total mass constant. Five particle-size gradation groups, denoted as Groups A–E, were defined according to the controlled particle-size fraction. Group A corresponds to the fraction smaller than 0.075 mm, Group B to 0.075–0.25 mm, Group C to 0.25–0.5 mm, Group D to 0.5–2 mm, and Group E to 2–5 mm. For each gradation group, the mass fraction of the controlled particle-size range was varied from 0% to 100% at intervals of 20%, generating cases such as A-0%, A-20%,  ..., and A-100%. The remaining particle-size fractions were mixed in equal proportions, with each fraction accounting for 0%, 5%, 10%, 15%, 20%, or 25%, depending on the controlled fraction. In total, 30 gradation cases were designed, as shown in Table 4.

**Table 4:** Particle size gradation cases.

ID	2-5 mm	0.5-2 mm	0.25-0.5 mm	0.075-0.25 mm	<0.075 mm
**A-0%**	25%	25%	25%	25%	0
**A-20%**	20%	20%	20%	20%	20%
**A-40%**	15%	15%	15%	15%	40%
**A-60%**	10%	10%	10%	10%	60%
**A-80%**	5%	5%	5%	5%	80%
**A-100%**	0	0	0	0	100%
**B-0%**	25%	25%	25%	0	25%
**B-20%**	20%	20%	20%	20%	20%
**B-40%**	15%	15%	15%	40%	15%
**B-60%**	10%	10%	10%	60%	10%
**B-80%**	5%	5%	5%	80%	5%
**B-100%**	0	0	0	100%	0
**C-0%**	25%	25%	0	25%	25%
**C-20%**	20%	20%	20%	20%	20%
**C-40%**	15%	15%	40%	15%	15%
**C-60%**	10%	10%	60%	10%	10%
**C-80%**	5%	5%	80%	5%	5%
**C-100%**	0	0	100%	0	0
**D-0%**	25%	0	25%	25%	25%
**D-20%**	20%	20%	20%	20%	20%
**D-40%**	15%	40%	15%	15%	15%
**D-60%**	10%	60%	10%	10%	10%
**D-80%**	5%	80%	5%	5%	5%
**D-100%**	0	100%	0	0	0
**E-0%**	0	25%	25%	25%	25%
**E-20%**	20%	20%	20%	20%	20%
**E-40%**	40%	15%	15%	15%	15%
**E-60%**	60%	10%	10%	10%	10%
**E-80%**	80%	5%	5%	5%	5%
**E-100%**	100%	0	0	0	0

### 2.3. Model and parameters

**Fig 1:**
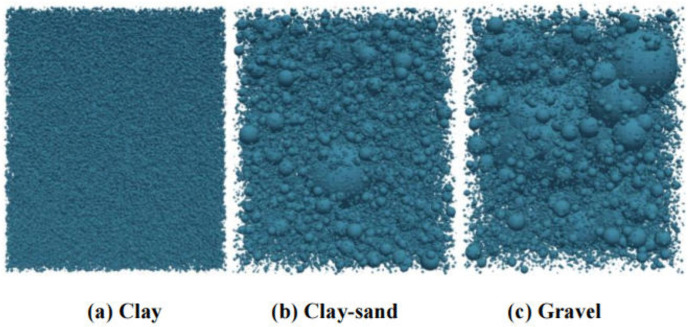
Schematic diagrams of particle composition characteristics. (a) Clay. (b) Clay-sand.(c) Gravel.

Particle modeling was conducted in PFC 3D according to the designed gradation cases to obtain the radius and position coordinates of all particles. The particle information was written into a Def file. The geometric model was defined using xml language, and the Def file was read by GenCase. The main program DualSPHysics was then used to read the file generated by GenCase, thereby outputting the particle model. The modeling image is shown in Fig 1.

The SPH main program DualSPHysics adopts the WCSPH model, where both the solid and liquid phases are simulated based on SPH and solved within the same framework, achieving direct coupling between the solid and liquid phases. The boundary condition used is the Crespo dynamic boundary condition, and the computational scheme of the DualSPHysics model employs the Symplectic numerical integration method. The karst cavity is generalized as a unit-length straight line-type 3D cavity with geometric dimensions of 50 cm × 5 cm × 5 cm. The two ends of the karst cavity are generalized as fluid boundaries, with a continuous water flow set at one end of the fluid boundary for 400 time steps. DualSPHysics operates with GPU acceleration. For post-processing, the Part VTK and Measure Tool codes are used. The output file of DualSPHysics is a  .bi4 binary file, which is processed by Part VTK to generate VTK, CSV, or ASCII files. These files are then visualized using ParaView. The Measure Tool can be used to interpolate and obtain the flow displacement, velocity, pressure, boundary force, and material loss amount for particles with corresponding IDs. The SPH computation parameters are shown in Table 5.

**Table 5:** DualSPHysics calculation parameters.

Numerical simulation parameters	Value	Numerical simulation parameters	Value
Particle spacing dp (m)	0.1	Number of particles Np	3072100
Smoothing kernel function	Cubic spline	Smoothing radius h (m)	0.0085
Pressure correction algorithm	δ-SPH algorithm	δ-SPH	0.1
Viscosity term	Artificial viscosity term	Artificial viscosity coefficient	0.05
$\rho_{\text{0}}$ (kg/m^3^)	1000	γ	7
Boundary conditions	Dynamic boundary conditions	Time integration scheme	Symplectic
CFL number	0.2	Physical simulation time (s)	4

### 2.4. Simulation of seepage erosion

**Fig 2:**
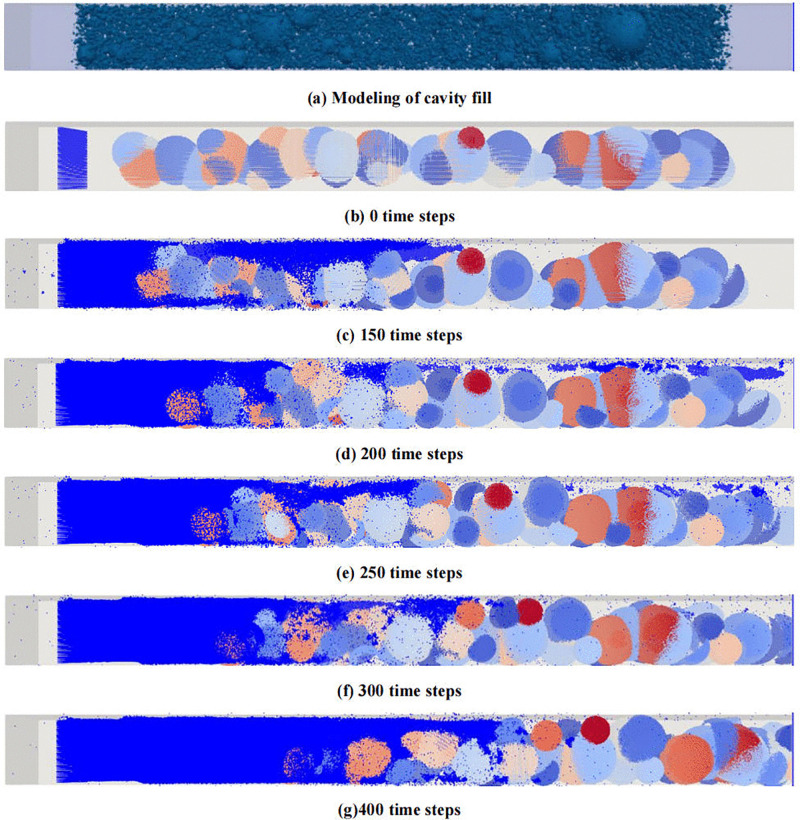
Evolution sequence of seepage-erosion-induced water inrush. (a) Modeling of cavity fill. (b) 0 time steps. (c) 150 time steps. (d) 200 time steps. (e) 250 time steps. (f) 300 time steps. (g) 400 time steps.

Fig 2 shows the simulated evolution of seepage-erosion-induced water inrush in a karst cavity. In Fig 2(a), the modeling of the cavity fillings is rendered using PFC 3D software, with particles of different sizes randomly distributed; Fig 2(b)–2(g) show the results of the water inrush process at different time steps, presented using Part VTK and Para View, where blue represents the water flow and the other circles represent particle bodies. The visualization post-processing is for illustrative purposes only. Initially, the filling structure is stable with minimal particle loss due to water erosion, but gradually the filling structure becomes unstable due to seepage erosion.

### 2.5. Validation of SPH model

The simulated development of seepage erosion, including initiation, acceleration, and stabilization, was compared with published physical model test observations. Zhang et al. reported that erosion is essentially a progressive process dominated by fine-particle migration and loss, resulting in cavity/loosened zones that evolve and then gradually converge to a stable state. This staged evolution is consistent with our SPH erosion curve trends [44].

**Fig 3:**
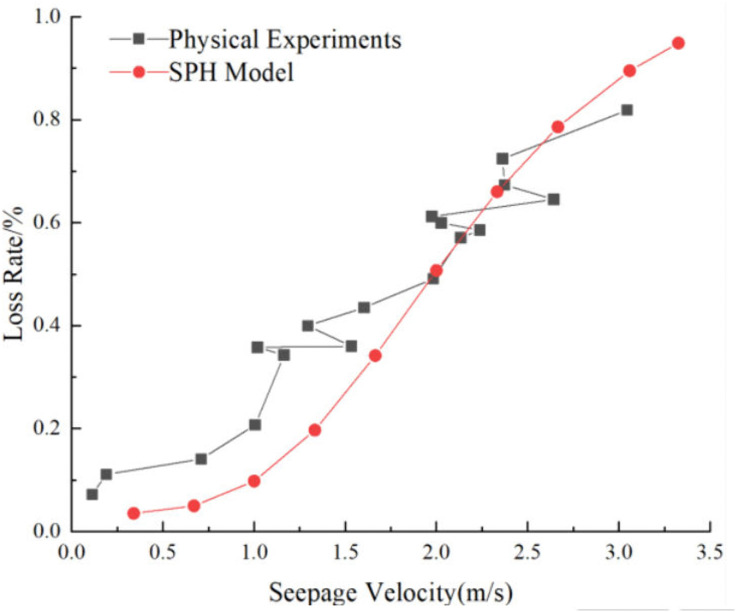
Comparison between physical experimental results and SPH simulation results.

Zhang experimentally established that the evolution of piping erosion can be quantitatively characterized by cumulative sand loss, flow conditions, and the development of internal preferential flow paths, which exhibit distinct stage transitions and eventual stabilization. Building on this physical experimental basis, our simulation parameters were configured to determine the correlation between seepage velocity and loss rate [45]. As shown in Fig 3, the results from our SPH model align well with the physical experimental data, validating the adopted parametric framework.

## 3. Effect of particle-size gradation

### 3.1. Particle loss rate

**Fig 4:**
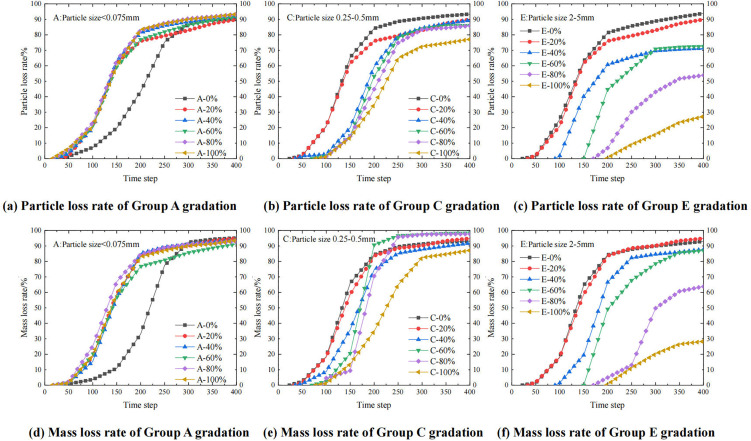
Particle loss rate and mass loss rate under different particle-size gradations. (a) Particle loss rate of Group A gradation. (b) Particle loss rate of Group C gradation. (c) Particle loss rate of Group E gradation. (d) Mass loss rate of Group A gradation. (e) Mass loss rate of Group C gradation. (f) Mass loss rate of Group E gradation.

Fig 4 shows the particle loss rate and mass loss rate of the cavity fill under different particle size distributions during water inrush. For the Group A gradation, particle loss rate and mass loss rate curves are similar. Under continuous water erosion, due to the lack of fine particles smaller than 0.075 mm in the fillings, the particle loss rate during the loss phase is significantly lower than that of other particle grading schemes. The trends in particle loss rate for the remaining A content levels are basically the same. When the water flow continues for 400 time steps, the particle loss rate exceeds 90% for all content levels, and the total loss rate increases slightly with increasing A content. This indicates that fine particles are lost early in the water inrush process. For the Group C gradation, there are some differences in the particle loss curves among the schemes. The particle loss processes for C-0% and C-20% are relatively fast. When the water flow continues for 400 time steps, there is minimal additional particle loss under the balanced loss state. Ultimately, the particle loss rate is highest for C-0% at 93%, and lowest for C-100% at 77%. In comparison, the particles are mostly coarse. Under continuous water erosion, the coarse particle skeleton gradually loosens, leading to instability of the overall particle structure and triggering a water inrush event. In the Group C gradation, there are significant differences in the particle loss curves among the schemes. As the content of E particles gradually increases, the loss curves for E-0% and E-20% in the early stages of loss are similar, with particles starting to loosen and lose as soon as the water flow begins to erode them. In the later stages, the particle loss rates differ significantly. When the water flow continues for 400 time steps, the particle loss rate reaches 94% for E-0%, 90% for E-20%, around 71% for E-40% and E-60%, 54% for E-80%, and 27% for E-100%. Comparing the particle loss chart and the mass loss chart, the coarsest particle size reaches 5 mm.

### 3.2. Loss rate

**Fig 5:**
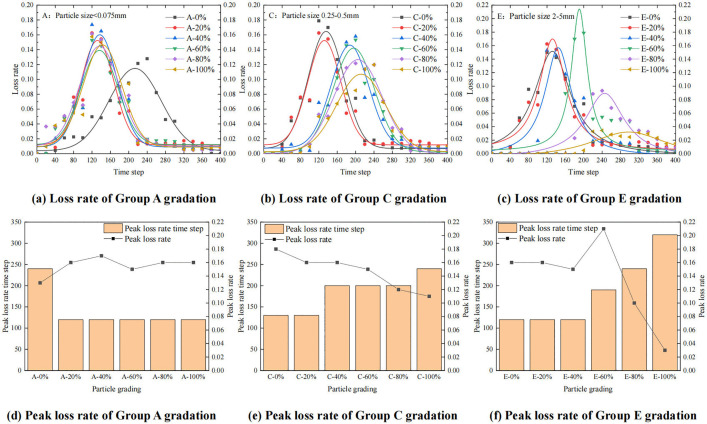
Loss rate and peak loss rate under different particle-size gradations. (a) Loss rate of Group A gradation. (b) Loss rate of Group C gradation. (c) Loss rate of Group E gradation. (d) Peak loss rate of Group A gradation. (e) Peak loss rate of Group C gradation. (f) Peak loss rate of Group E gradation.

To fully discuss the impact of particle size distribution on water inrush in a dissolved cavity, Fig 5 presents the particle loss rate, which records the change in particle loss rate every 20 time steps. The Gaussian function is used to fit the data and analyze the variation pattern of the loss rate. In Group A gradation, under the continuous flow of water, the particle loss rate gradually increases to a peak and then gradually decreases until the particle loss ends. The particle loss rate of A-0% is significantly different from that of other compositions, with a maximum loss rate of 0.13, reaching its peak at the 240 time steps. The particle loss rates of the five compositions from A-20% to A-100% all reach their peaks around the 120^th^ time step. The maximum loss rates are 0.16 for A-20%, 0.17 for A-40%, 0.15 for A-60%, 0.16 for A-80%, and 0.16 for A-100%. The content of fine particles has a relatively small impact on the loss rate. In Group C gradation, when the content of 0.25–0.5 mm particles is low, the particle loss rate is faster. As the content of 0.075–0.25 mm particles increases to over 40%, the time to reach the peak loss rate gradually delays, and the particle loss rate also gradually decreases. In E particle size scheme, as the content of 2–5 mm particles gradually increases, the particle loss rate of E-80% is much lower than that of other compositions, indicating that the filling medium composed of the particle size distribution in the E-80% case is relatively stable and less prone to water inrush.

## 4. Effect of seepage velocity

### 4.1. Particle loss rate

**Fig 6:**
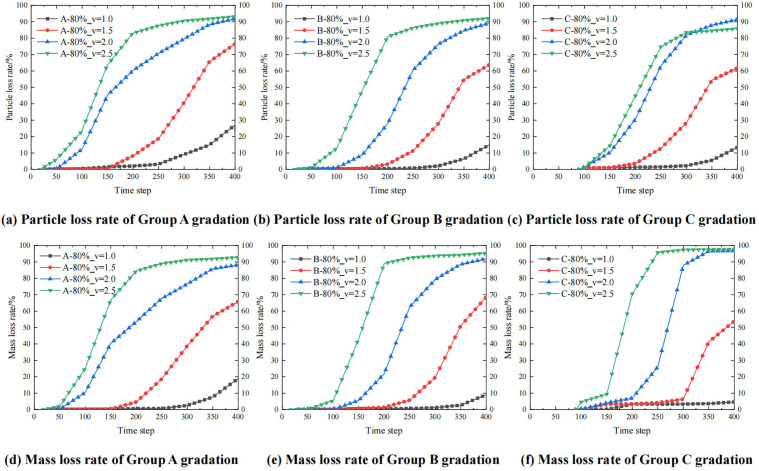
Particle loss rate and mass loss rate under different seepage velocities. (a) Particle loss rate of Group A gradation. (b) Particle loss rate of Group B gradation. (c) Particle loss rate of Group C gradation. (d) Mass loss rate of Group A gradation. (e) Mass loss rate of Group B gradation. (f) Mass loss rate of Group C gradation.

Discuss the characteristics of particle loss in a dissolved cavity under different inrush flow velocities. Three particle size schemes are selected, with seepage velocities controlled at 1.0 m/s, 1.5 m/s, 2.0 m/s, and 2.5 m/s, respectively. Water erosion is simulated for 400 time steps, as shown in Table 6. To simplify, only the particle loss rate and mass loss rate for the A-80%, B-80%, and C-80% cases under different seepage velocities are presented in Fig 6. When the seepage velocity is low at 1.0 m/s, the A-80% case shows signs of loss at the 150^th^ time step, with a particle loss rate of 27% at the 400 time steps; the B-80% scheme shows signs of loss at the 300 time steps, with a final particle loss rate of 13%; the C-80% scheme has a final particle loss rate of 12%. When the seepage velocity is 1.5 m/s, the final particle loss rates are 77% for A-80%, 62% for B-80%, and 28% for C-80% case with only 7% mass loss mostly due to fine particle loss before the 300 time steps. When the seepage velocity is 2.0 m/s, the particle loss rate accelerates, increasing by over 40% at the 150 time steps, and then slightly decreases after the 250 time steps, with a final particle loss rate of around 90%, with mostly coarse particles lost in the mid to late stages of loss.

**Table 6:** Particle-size gradation and seepage velocity cases (i = 20, 40, 60, 80).

Seepage rate (m/s)	<0.075 mm (A-i%)	0.075-0.25 mm(B-i%)	0.25-0.5 mm(C-i%)
1.0	A-i%_v = 1.0	B-i%_ v = 1.0	C-i%_ v = 1.0
1.5	A-i%_ v = 1.5	B-i%_ v = 1.5	C-i%_ v = 1.5
2.0	A-i%_ v = 2.0	B-i%_ v = 2.0	C-i%_ v = 2.0
2.5	A-i%_ v = 2.5	B-i%_ v = 2.5	C-i%_ v = 2.5

### 4.2. Loss rate

**Fig 7:**
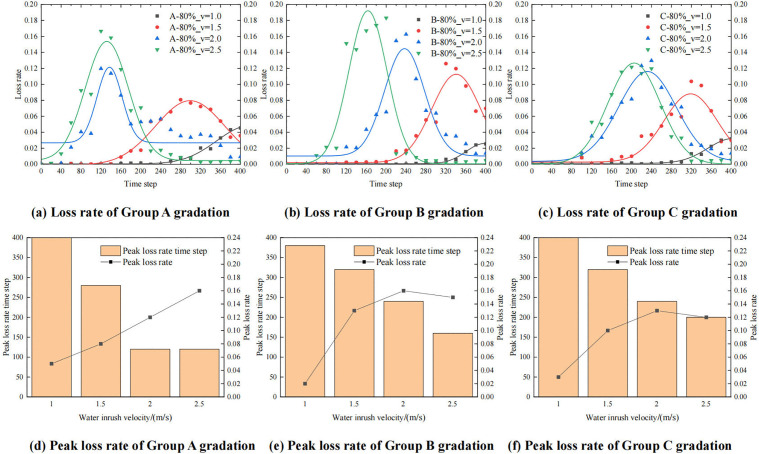
Loss rate and peak loss rate under different seepage velocities. (a) Loss rate of Group A gradation. (b) Loss rate of Group B gradation. (c) Loss rate of Group C gradation. (d) Peak loss rate of Group A gradation. (e) Peak loss rate of Group B gradation. (f) Peak loss rate of Group C gradation.

Fig 7 presents the incremental particle loss rate and peak incremental particle loss rate of the A-80%, B-80%, and C-80% cases under different seepage velocities. For the A-80% case, when the velocity is 1.0 m/s, the loss rate increases at the 240 time steps, with a maximum of 0.05. At a velocity of 1.5 m/s, the loss rate peaks at 0.08 at the 280^th^ time step and slightly decreases after the 340 time steps. When the seepage velocity is 2.0 m/s, the loss rate peaks at the 120 time steps, with a maximum particle loss rate of 0.12, and remains around 0.05 after the 180 time steps. At a velocity of 2.5 m/s, the maximum particle loss rate is 0.16, which decreases to 0.07 after the 180 time steps and to 0.01 after the 320 time steps. As the velocity increases, the maximum difference in peak loss rates is 0.11. For the B-80% case, when the velocity is 1.0 m/s, the loss rate increases at the 320 time steps, with a maximum of 0.02. At a velocity of 1.5 m/s, the loss rate is 0.05 at the 300 time steps and peaks at 0.13 at the 320 time steps. When the velocity is 2.0 m/s, the loss rate increases sharply at the 220 time steps and reaches its peak of 0.16 at the 240 time steps. At a velocity of 2.5 m/s, the maximum particle loss rate is 0.18. As the seepage velocity increases, the peak loss rate is highest at 2.5 m/s, with a maximum difference in peak loss rates of 0.14. For the C-80% case, when the velocity is 1.0 m/s, the loss rate increases at the 320 time steps, with a maximum of 0.03. At a velocity of 1.5 m/s, the loss rate peaks at 0.1 at the 320 time steps. When the velocity is 2.0 m/s, the loss rate reaches its peak at the 240 time steps, with a maximum particle loss rate of 0.13. At a velocity of 2.5 m/s, the maximum particle loss rate is 0.12, and the loss rate gradually slows down in the later stages. As the velocity increases, the maximum difference in peak loss rates is 0.09.

## 5. Effect of cavity confining stress

### 5.1. Particle loss rate

The particle-loss characteristics under different cavity confining stresses were analyzed. Seven confining stress schemes are selected, with confining stresses of 0 MPa, 2 MPa, 3 MPa, 4 MPa, 6 MPa, 8 MPa, and 10 MPa, respectively. Seepage erosion is simulated for 400 time steps, and the cases are presented in Table 7.

**Table 7:** Particle-size gradation and confining stress cases (i = 20, 40, 60, 80).

Confining stress (MPa)	<0.075 mm(A-i%)	0.075-0.25 mm(B-i%)	0.25-0.5 mm(C-i%)
**0**	A-i%_0 MPa	B-i%_0 MPa	C-i%_0 MPa
**2**	A-i%_2 MPa	B-i%_2 MPa	C-i%_2 MPa
**3**	A-i%_3 MPa	B-i%_3 MPa	C-i%_3 MPa
**4**	A-i%_4 MPa	B-i%_4 MPa	C-i%_4 MPa
**6**	A-i%_6 MPa	B-i%_6 MPa	C-i%_6 MPa
**8**	A-i%_8 MPa	B-i%_8 MPa	C-i%_8 MPa
**10**	A-i%_10 MPa	B-i%_10 MPa	C-i%_10 MPa

**Fig 8:**
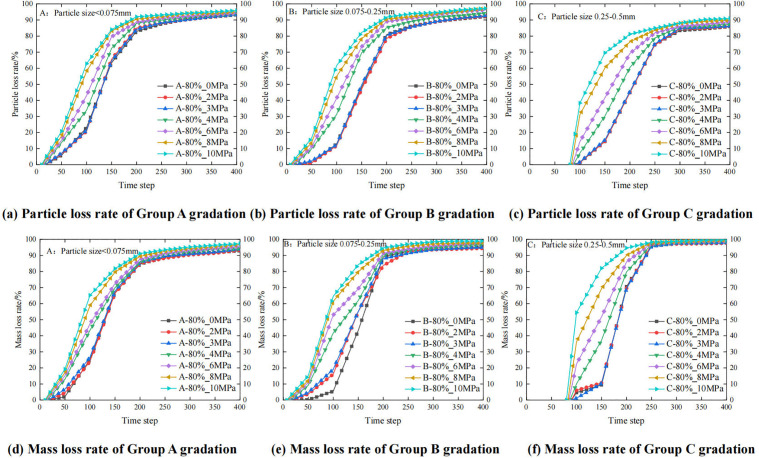
Particle loss rate and Mass loss rate under different confining stresses. (a) Particle loss rate of Group A gradation. (b) Particle loss rate of Group B gradation. (c) Particle loss rate of Group C gradation. (d) Mass loss rate of Group A gradation. (e) Mass loss rate of Group B gradation. (f) Mass loss rate of Group C gradation.

For clarity, only the particle loss rate and mass loss rate for the A-80%, B-80%, and C-80% cases under different confining stresses of the karst cavity are shown in Fig 8. For all three cases, the application of 2 MPa and 3 MPa confining stress has no significant effect on the overall loss pattern, and the loss curves are basically similar and overlap. When the confining stress increases to 4 MPa, its influence on the particle loss rate becomes significantly more pronounced. Particle loss occurs immediately after the onset of water erosion. In the particle loss phase, from time step 0–200, the particle loss rate increases by approximately 8% for the A-80% case, 20% for the B-80% case, and 60% for the C-80% case, compared to the scenario without confining stress, for the same duration of loss. When the confining stress is 10 MPa, the particle loss rate during the erosion process increases by 50%, 60%, and 75% for the A-80%, B-80%, and C-80%, respectively, compared to the scenario without confining stress, for the same duration of loss. By comparing the particle loss and mass loss diagrams, as the applied confining stress increases, the difference between the particle loss rate and mass loss rate for the A-80% is small, indicating that as the content of fine particles in the fill material increases, the influence of confining stress on particle loss decreases. When the confining stress is low, the initial mass loss rate is higher than the particle loss rate for the B-80% grading scheme, indicating that coarser particles are lost initially. The total mass loss rate for the C-80% is close to 100%, but the particle loss rate is around 90%, indicating that when the proportion of coarse particles is high, the application of confining stress accelerates the loss of larger particles.

### 5.2. Loss rate

**Fig 9:**
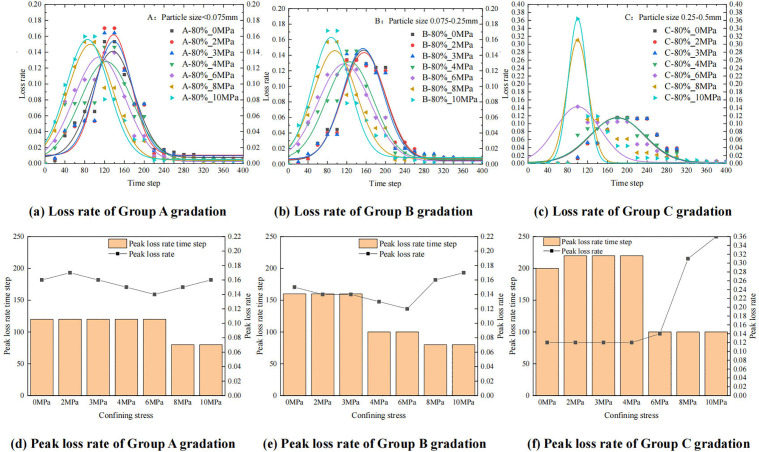
Loss rate and peak loss rate under different confining stresses. (a) Loss rate of Group A gradation. (b) Loss rate of Group B gradation. (c) Loss rate of Group C gradation. (d) Peak loss rate of Group A gradation. (e) Peak loss rate of Group B gradation. (f) Peak loss rate of Group C gradation.

Fig 9 presents the erosion rate and peak erosion rate of the A-80%, B-80%, and C-80% cases under different confining stresses in the karst cavity. For the A-80% case, under continuous water flow, the particle loss rate gradually increases to a peak and then gradually decreases until the erosion process ends. As the confining stress increases from 0 MPa to 10 MPa, the loss rate of the coarsest particles remains around 0.16, indicating that the increase in confining stress has no effect on the maximum erosion rate. When a confining stress of 10 MPa is applied, the peak loss rate is reached at the 80 time steps, which is 70 time steps earlier than when no confining stress is applied. As shown in the Fig 9, when the content of particles smaller than 0.075 mm reaches 80%, the magnitude of the confining stress does not affect the maximum erosion rate but only accelerates the overall loss process. For the B-80% case, when the applied confining stress is relatively low, at 2 MPa and 3 MPa, the loss rate curves are similar to that without confining stress, with a maximum loss rate of 0.14 reached at the 160 time steps. As the confining stress increases, the time to reach the peak loss rate decreases, and the maximum loss rate also decreases: the maximum loss rate is 0.13 at 4 MPa and 0.12 at 6 MPa, both reached between the 100 and 140 time steps. At 8 MPa, the maximum loss rate increases again to 0.16, reached at the 80 time steps; at 10 MPa, the maximum loss rate is 0.17, also reached at the 80 time steps. Overall, the confining stress has an impact on the loss rate when it is between 4 MPa and 6 MPa. For the C-80% particle grading scheme, as the proportion of coarse particles increases, the particle erosion rate differs significantly from the previous schemes: as the confining stress gradually increases, the time to reach the peak loss rate becomes shorter. The loss rate curves remain similar as the confining stress increases up to 4 MPa: the peak loss rate is reached at the 220 time steps, with a maximum loss rate of around 0.12. When the confining stress increases to 6 MPa, the maximum loss rate increases, reaching a peak of 0.14 at the 100 time steps. At 8 MPa, the maximum loss rate rises rapidly to 0.31, and at 10 MPa, the maximum erosion rate is 0.36, both reached at the 100 time steps.

## 6. Catastrophe mechanism of water inrush

Particle-size gradation, confining stress, and seepage velocity are the three key factors controlling the evolution of sudden inrush water disasters. Particle grading is the intrinsic factor causing seepage erosion-induced inrush water, while confining stress in the karst cavity and seepage velocity are extrinsic factors. The combined effect of intrinsic and extrinsic factors leads to the occurrence of inrush water. Therefore, we will discuss the changes in particle loss rate and mass loss rate during the evolution of seepage erosion-induced inrush water under two conditions: Particle-size gradation-seepage velocity and p Particle-size gradation-confining stress aiming to identify the sudden change point in the loss rate as an indicative signal for determining the onset of inrush water disasters.

### 6.1. Particle-size gradation – Seepage velocity

#### 6.1.1. Group A gradation – Seepage velocity.

**Fig 10:**
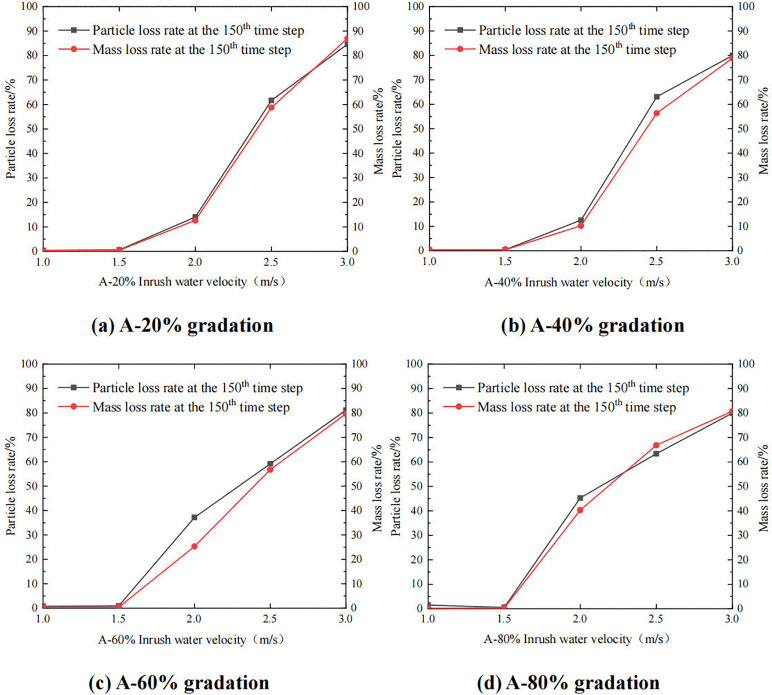
Relationship between seepage velocity and particle loss rate for Group A gradation. (a) A-20% gradation. (b) A-40% gradation. (c) A-60% gradation. (d) A-80% gradation.

By selecting different contents of Group A gradation, the particle loss rate at the 150 time steps was recorded. Fig 10 shows the relationship between particle loss rate and seepage velocity for seepage-erosion-induced water inrush in a karst cavity. For the A-20% and A-40% cases, the particle loss rate and mass loss rate curves increase in the same direction. As the seepage velocity increases to 2.0 m/s, the particle loss rate changes slightly and is less affected by the seepage velocity. When the seepage velocity exceeds 2.0 m/s, the particle loss rate increases in a downward-opening parabolic manner, with a higher seepage velocity resulting in a higher particle loss rate. For the A-60% and A-80% cases, the particle loss rate increases rapidly under the influence of seepage velocity. As the seepage velocity increases to 1.5 m/s, the particle loss rate remains unchanged, indicating that the filler medium particles are less affected by the seepage velocity. When the seepage velocity exceeds 1.5 m/s, the particle loss rate in the A-60% case increases linearly, with a higher seepage velocity resulting in a higher particle loss rate.

Analysis shows that: For Group A gradation with a content less than 40%, the seepage velocity of around 2.0 m/s is the critical point. For Group A gradation with a content greater than 60%, the seepage velocity of around 1.5 m/s is the critical point. When the seepage velocity is below the critical point, changes in seepage velocity do not affect the loss of filler medium particles. When the seepage velocity exceeds the critical point, the particle loss rate increases rapidly, with a higher seepage velocity resulting in more particle loss. The effect of seepage velocity on particle loss is significantly influenced by the content of Group A gradation. As the content of Group A gradation increases, the critical point of seepage velocity also increases.

#### 6.1.2. Group B gradation – Seepage velocity.

**Fig 11:**
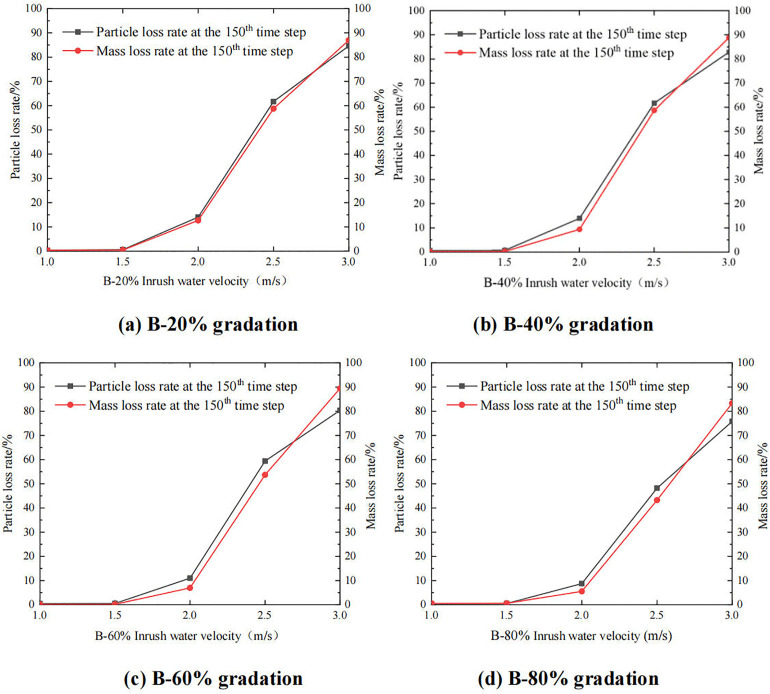
Relationship between seepage velocity and particle loss rate for Group B gradation. (a) B-20% gradation. (b) B-40% gradation. (c) B-60% gradation. (d) B-80% gradation.

By selecting different contents of Group B gradation, the particle loss rate at the 100 time steps was recorded. The curve of particle loss rate corresponding to seepage erosion-induced inrush water in the karst cavity under the influence of seepage velocity is shown in Fig 11. For the B-40% case, the particle loss rate and mass loss rate curves increase in the same direction under the influence of seepage velocity. As the seepage velocity increases to 2.0 m/s, the particle loss rate changes slightly. When the seepage velocity exceeds 2.0 m/s, the particle loss rate increases in a downward-opening parabolic manner, with a higher seepage velocity resulting in a higher particle loss rate. For the B-60% and B-80% cases, the particle loss rate increases rapidly under the influence of seepage velocity. As the seepage velocity increases to 2.0 m/s, the particle loss rate changes slightly. When the seepage velocity exceeds 2.0 m/s, the particle loss rate for the B-80% case increases linearly, with a higher seepage velocity resulting in a higher particle loss rate.

The results show the seepage velocity of 2.0 m/s is the critical point. When the seepage velocity is below 2.0 m/s, changes in seepage velocity do not affect the loss of filler medium particles. When the seepage velocity exceeds 2.0 m/s, the growth rate of particle loss transitions from a downward-opening parabolic pattern to a linear pattern, with a higher loss rate resulting in more particle loss. The effect of seepage velocity on particle loss is not influenced by the content of Group B gradation.

#### 6.1.3. Group C gradation – Seepage velocity.

**Fig 12:**
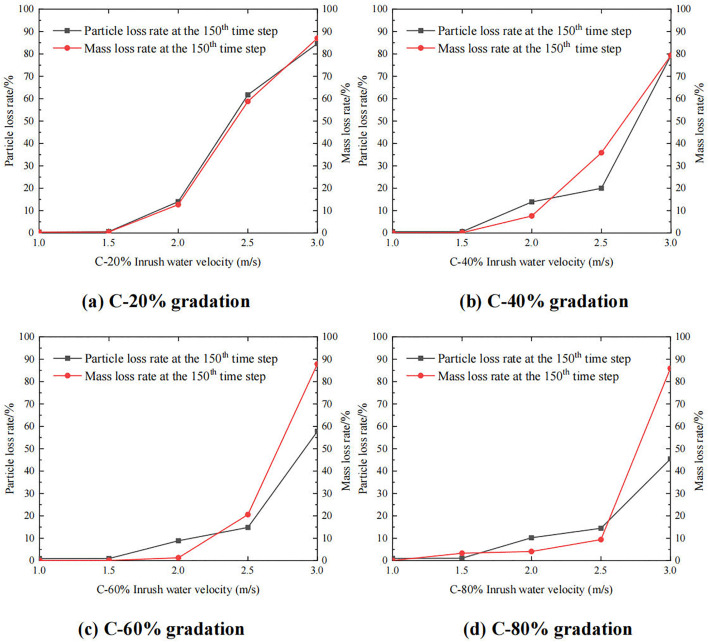
Relationship between seepage velocity and particle loss rate for Group C gradation. (a) C-20% gradation. (b) C-40% gradation. (c) C-60% gradation. (d) C-80% gradation.

By selecting different contents of Group C gradation, the particle loss rate at the 150 time steps was recorded. Fig 12 shows the relationship between particle loss rate and seepage velocity. For the C-40% cases, the mass loss rate increases rapidly under the influence of seepage velocity. As the seepage velocity increases to 2.5 m/s, the particle loss rate changes slightly. When the seepage velocity exceeds 2.5 m/s, the particle loss rate increases linearly, with a higher seepage velocity resulting in a higher particle loss rate. For the C-60% and C-80% cases, the mass loss rate increases rapidly under the influence of seepage velocity. As the seepage velocity increases to 2.5 m/s, the particle loss rate remains unchanged. When the seepage velocity exceeds 2.5 m/s, the particle loss rate increases linearly, with a higher seepage velocity resulting in a higher particle loss rate.

Analysis shows that: For Group C gradation with a content less than 20%, the seepage velocity of around 2.0 m/s is the critical point. For Group C gradation with a content greater than 40%, the seepage velocity of around 2.5 m/s is the critical point. When the seepage velocity is below the critical point, changes in seepage velocity do not affect the loss of filler medium particles. When the seepage velocity exceeds the critical point, the particle loss rate increases rapidly, with a higher seepage velocity resulting in more particle loss. The effect of seepage velocity on particle loss is significantly influenced by the content of Group C gradation. As the content of Group C gradation increases, the critical point of seepage velocity also increases.

### 6.2. Particle-size gradation and confining stress

#### 6.2.1. Group A gradation- Confining stress.

**Fig 13:**
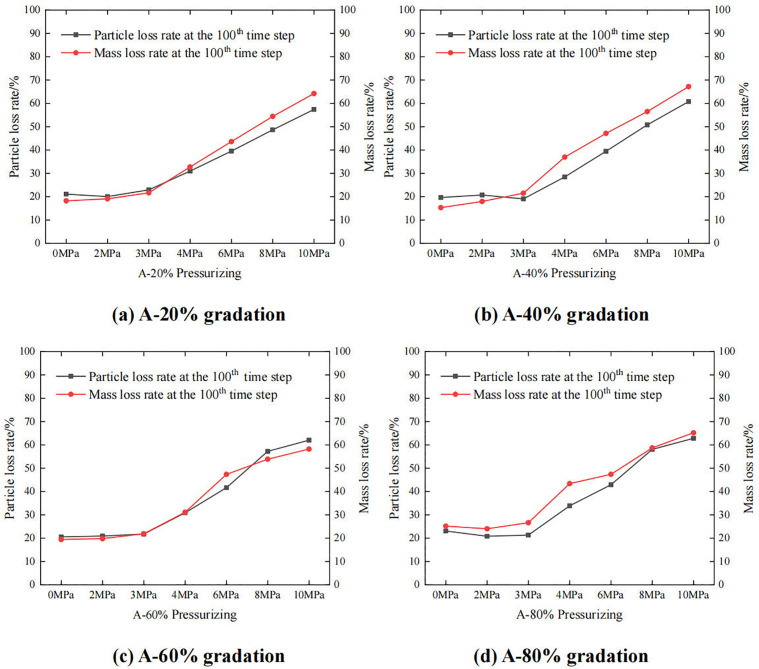
Relationship between confining stress and particle loss rate for Group A gradation. (a) A-20% gradation. (b) A-40% gradation. (c) A-60% gradation. (d) A-80% gradation.

By selecting different contents of Group A gradation, the particle loss rate at the 100 time steps was recorded. Fig 13 shows the relationship between particle loss rate and confining stress. For the A-20% and A-40% cases, the particle loss rate and mass loss rate curves show consistent growth trends under the influence of confining stress. When the applied confining stress is 2 MPa and 3 MPa, the particle loss rate remains unchanged, indicating that the filler particles are less affected by the confining stress. As the confining stress on the karst cavity wall exceeds 3 MPa, the particle loss rate increases linearly, with a higher confining stress resulting in a higher particle loss rate. For the A-60% and A-80% cases, the particle loss rate and mass loss rate curves also show consistent growth trends under the influence of confining stress. When the applied confining stress is 2 MPa and 3 MPa, the particle loss rate remains unchanged. As the confining stress exceeds 3 MPa, the particle loss rate increases in a downward-opening parabolic manner, with a higher confining stress resulting in a higher particle loss rate. Under the same particle size gradation, the filler medium is significantly affected by the confining stress. When the confining stress exceeds 8 MPa, the growth trend of the particle loss rate slows down, and the impact of increasing confining stress on particle loss decreases.

Analysis shows that confining stress is less than 3 MPa, changes in confining stress do not affect the loss of filler particles. When the confining stress exceeds 3 MPa, the particle loss rate increases linearly, with a higher confining stress resulting in more particle loss. The effect of confining stress on particle loss is not influenced by the content of Group A gradation.

#### 6.2.2. Group B gradation – Confining stress in karst cavity.

**Fig 14:**
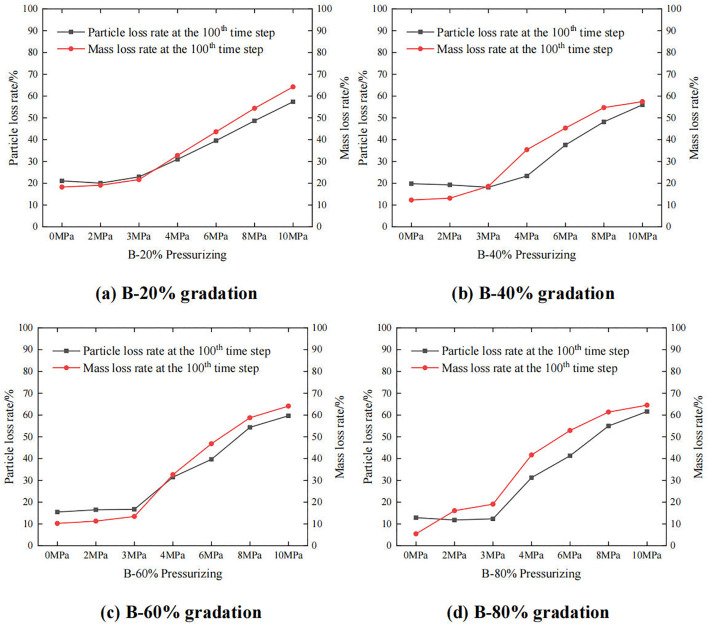
Relationship between confining stress and particle loss rate for Group B gradation. (a) B-20% gradation. (b) B-40% gradation. (c) B-60% gradation. (d) B-80% gradation.

By selecting consists of Group B gradation, and the particle loss rate corresponding to 100 time steps is recorded. Fig 14 shows the relationship between particle loss rate and confining stress. For the B-40% case, the mass loss rate curve increases rapidly under the influence of confining stress, with coarse particles in the B-40% the first to erode. When the applied confining stress is 2 MPa, 3 MPa, or 4 MPa, the particle loss rate remains unchanged. As the confining stress exceeds 4 MPa, the particle loss rate increases in a downward-opening parabolic manner, with higher confining stress leading to higher particle loss rates. The filler medium in the B-40% is significantly affected by confining stress. For the B-60% and B-80% cases, the mass loss rate curve also increases rapidly under the influence of confining stress, with coarse particles in the B-80% the first to erode. When the applied confining stress is 2 MPa or 3 MPa, the particle loss rate remains unchanged. As the confining stress exceeds 3 MPa, the particle loss rate increases in a downward-opening parabolic manner, with higher confining stress leading to higher particle loss rates.

The results indicate that the B-40% case undergoes a sudden change at a confining stress of around 4 MPa, whereas cases with a Group B mass fraction greater than 60% undergoes a sudden change at a confining stress of around 3 MPa. When the confining stress is less than the point of sudden change, changes in confining stress do not affect the particle loss of the filler medium. When the confining stress exceeds the point of sudden change, the particle loss rate increases in a parabolic manner, with higher confining stress leading to more particle loss. The effect of confining stress on particle loss is relatively insensitive to the content of the Group B gradation.

#### 6.2.3. Group C gradation – Confining stress in karst cavity.

**Fig 15:**
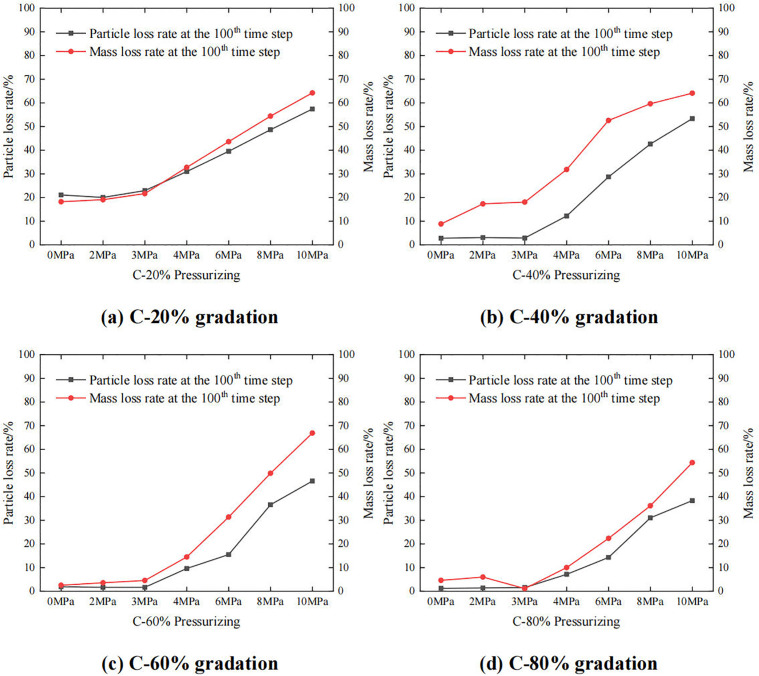
Relationship between confining stress and particle loss rate for Group C gradation. (a) C-20% gradation. (b) C-40% gradation. (c) C-60% gradation. (d) C-80% gradation.

Cases with different mass fractions of Group C particles were selected as the cavity fill, and the cumulative particle loss rate at 100 time steps was recorded. Fig 15 shows the corresponding relationship between particle loss rate and confining stress. For the C-40% case, the mass loss rate under the influence of confining stress is significantly greater than the particle loss rate, with coarse particles being the first to erode under the C-40% case. With confining stresses of 2 MPa and 3 MPa applied, the particle loss rate increases slowly. As the confining stress on the cavity wall exceeds 3 MPa, the particle loss rate exhibits a downward-opening linear growth, with a higher particle loss rate at greater confining stresses, indicating that the fill medium under the C-40% case is significantly affected by confining stress. For the C-60% and C-80% cases, the particle loss rate and mass loss rate curves under the influence of confining stress grow in the same direction, with coarse particles eroding first as the confining stress increases. With confining stresses of 2 MPa, 3 MPa, 4 MPa, and 6 MPa applied, the particle loss rate increases slowly. As the confining stress exceeds 6 MPa, the particle loss rate exhibits an upward-opening parabolic growth, with a higher particle loss rate at greater confining stresses.

These results demonstrate a clear threshold behavior in confining-stress-controlled particle loss. For the C-40% case, the particle loss rate changes markedly at approximately 3 MPa; for cases with a Group C mass fraction greater than 60%, the transition shifts to a higher stress level of approximately 6 MPa. When the applied confining stress remains below the mutation point, the granular skeleton maintains a relatively stable load-bearing structure, and particle loss is primarily governed by seepage-driven migration, showing limited sensitivity to confinement. In contrast, once the confining stress exceeds the mutation point, confinement no longer plays a purely stabilizing role; instead, it promotes structural degradation and accelerated loss. The particle loss rate increases nonlinearly with confining stress, implying that high stress amplifies local stress concentration and facilitates contact-network collapse after fine-particle depletion. Importantly, the mutation point rises with increasing C content, indicating that a larger Group C fraction enhances the initial skeleton resistance against confinement-induced instability, but once destabilization is triggered, the loss rate escalates rapidly with further stress increase. Physical laboratory tests by other scholars have revealed distinct mechanisms under varying confining stresses [46]. Under low confining stress, the contact network maintains stable load-transfer paths, facilitating gradual particle rearrangement and densification—behavior consistent with the “slow development” stage. In contrast, under high confining stress, the coarse-grained skeleton is subjected to stronger compressive constraints, and seepage drag becomes concentrated around preferential channels. Once fine particles are eroded and voids form, the skeleton becomes highly susceptible to local buckling or breakage, leading to a sudden acceleration of soil loss. Accordingly, in our SPH numerical simulation, an open boundary condition was set at the outburst outlet to replicate the physical process wherein water pressure is instantly released upon gushing into the tunnel.

## 7. Early -warning indicators for water-inrush catastrophe

**Fig 16:**
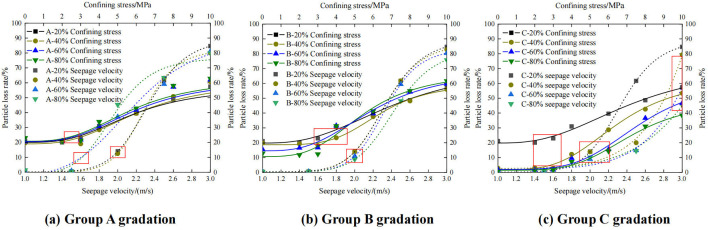
Critical transition region of particle loss under particle-size gradation, seepage velocity, and confining stress. (a) Group A gradation. (b) Group B gradation. (c) Group C gradation.


Logistic regression was used to fit the particle loss rate curves under the combined effects of particle-size gradation, seepage velocity, and confining stress. The fitted curves satisfy Equation (1). The parameters, including those denoted as ${\text{K}}_{\text{1}}$, ${\text{K}}_{\text{2}}$, ${\text{K}}_{\text{3}}$, P and R^2^ for different Group A gradation shown in Table 8. The fitted curve of the particle loss rate at the sudden change point of the loss rate is illustrated in Fig 16. By taking the second derivative of the fitted curve function, the first derivative satisfies Equation (2), and the second derivative satisfies Equation (3). The inflection point of the fitted curve represents the sudden change point where the particle loss rate accelerates, and the corresponding particle loss rate can serve as an early warning criterion indicator for seepage erosion-induced inrush water catastrophe in the cavity.

**Table 8:** Logistic regression parameters for the fitted particle loss rate curves.

Grading No.	Early warning indicator	K1		K2		K3		P		R^2^
		Value	Standard deviation	Value	Standard deviation	Value	Standard deviation	Value	Standard deviation	
**A-20%**	Seepage velocity	0.0017	0.0024	0.8961	0.0054	2.329	0.0048	11.1649	0.1931	0.975
**A-40%**		0.002	0.0009	0.8222	0.0017	2.2831	0.0019	13.0746	0.0907	0.985
**A-60%**		0.0078	1.4359	0.811	3.5632	2.0865	4.9332	3	22.4784	0.897
**A-80%**		0.0053	1.8619	0.7993	2.8585	1.8847	3.087	3	23.367	0.964
**A-20%**	Confining stress	0.1979	0.0175	0.732	0.1675	7.3271	1.9006	2.6354	0.8095	0.942
**A-40%**		0.1904	0.0465	0.6075	0.2231	6.0734	2.6526	3	2.3946	0.921
**A-60%**		0.2055	0.0544	0.6203	0.2439	5.9337	2.8903	3	2.7335	0.918
**A-80%**		0.2081	0.0569	0.6282	0.2344	5.7632	2.7122	3	2.7154	0.927
**B-20%**	Seepage velocity	0.0017	0.0024	0.8961	0.0054	2.3290	0.0048	11.1649	0.1931	0.976
**B-40%**		0.0032	0.0022	0.8694	0.0047	2.3145	0.0045	11.5313	0.1874	0.939
**B-60%**		0.0035	0.0014	0.8367	0.0028	2.3296	0.0027	12.606	0.1362	0.952
**B-80%**		0.0028	0.0012	0.829	0.0035	2.4289	0.0028	11.1815	0.1348	0.957
**B-20%**	Confining stress	0.1979	0.0175	0.732	0.1675	7.3271	1.9006	2.6354	0.8095	0.964
**B-40%**		0.1867	0.0107	0.6003	0.043	6.3496	0.3952	4.5454	1.0117	0.967
**B-60%**		0.1454	0.0326	0.6956	0.1507	6.004	1.3482	3.012	1.2592	0.951
**B-80%**		0.1071	0.0409	0.6762	0.1308	5.4634	1.0388	3.3238	1.4907	0.969
**C-20%**	Seepage velocity	0.0017	0.0024	0.8961	0.0054	2.329	0.0048	11.1649	0.1931	0.976
**C-40%**		0.0268	0.0713	1881.9	1.84E7	8.7707	11842	7.2818	16.2345	0.917
**C-60%**		0.0087	0.9376	0.5771	7.5984	2.668	22.261	3	36.7956	0.902
**C-80%**		0.0182	0.0498	1511.7	1.65E7	12.159	22819	5.8311	11.2411	
**C-20%**	Confining stress	0.1979	0.0175	0.732	0.1675	7.3271	1.9006	2.6354	0.8095	
**C-40%**		0.0187	0.0146	0.6373	0.0771	6.524	0.5561	3.601	0.7061	
**C-60%**		0.02	0.0203	0.6241	0.2042	7.6727	1.463	4.1068	1.682	
**C-80%**		0.0143	0.0127	0.4822	0.0953	7.2359	0.8372	4.257	1.2634	

$$\begin{array}{c} y=K_{\text{2}}+\frac{\left(K_{\text{1}}-K_{\text{2}}\right)}{\left(\text{1}+{\left(\frac{x}{K_{\text{3}}}\right)}^{P}\right)} \end{array}$$ (1)

where ${\text{K}}_{\text{1}}$, ${\text{K}}_{\text{2}}$, ${\text{K}}_{\text{3}}$, and P are coefficients.


$$\begin{array}{c} y^{\prime }=\frac{\frac{P\left(K_{\text{1}}-K_{\text{2}}\right)}{K_{\text{3}}^{P}x^{P-\text{1}}}}{{\left(\text{1}+\frac{x^{P}}{K_{\text{3}}^{P}}\right)}^{\text{2}}} \end{array}$$ (2)

### 7.1. Group A gradation

For the Group A gradation, the particle loss rate exhibits a gradual increase with increasing confining stress. The sudden change point of the particle loss rate corresponds to a confining stress of 2.6 MPa, with a particle loss rate reaching 24%. The particle loss rate shows a sudden increase with increasing seepage velocity. When the content of the Group A gradation is less than 60%, the sudden change point of the particle loss rate corresponds to a seepage velocity of 2.0 m/s, with a particle loss rate reaching 13%. When the content of the Group A gradation is greater than 60%, the sudden change point corresponds to a seepage velocity of 1.6 m/s, with a particle loss rate reaching 8%. When both the seepage velocity and confining stress are below their respective sudden change points, particle loss is not affected by these two factors. Conversely, as the seepage velocity and confining stress increase, the particle loss rate grows linearly and rapidly, leading to greater particle loss. When the seepage velocity exceeds 2.5 m/s and the confining stress exceeds 8 MPa, the influence of seepage velocity and confining stress on particle loss gradually diminishes.


$$\begin{array}{c} y^{\prime \prime }=\frac{\frac{\left(K_{\text{1}}-K_{\text{2}}\right)P}{K_{\text{3}}^{P}}x^{P-\text{1}}\left(\frac{\text{2}P}{K_{\text{3}}^{\text{2}P}}x^{\text{2}P-\text{1}}+\frac{\text{2}P}{K_{\text{3}}^{P}}x^{P-\text{1}}\right)-\frac{\left(K_{\text{1}}-K_{\text{2}}\right)P(P-\text{1})}{K_{\text{3}}^{P}}{\left(\text{1}+\frac{x^{P}}{K_{\text{3}}^{P}}\right)}^{\text{2}}x^{P-\text{2}}}{{\left(\text{1}+\frac{x^{P}}{K_{\text{3}}^{P}}\right)}^{\text{4}}} \end{array}$$ (3)

### 7.2. Group B gradation

For the Group B gradation, the particle loss rate increases abruptly with increasing seepage velocity. The inflection point for the particle loss rate corresponds to a seepage velocity of 2.0 m/s, with a particle loss rate of 14%. When the content of the Group B gradation is less than 40%, the particle loss rate increases gradually with increasing confining stress. The inflection point for the particle loss rate corresponds to a confining stress of 4.02 MPa, with a particle loss rate of 23%. When the content of the Group B gradation is greater than 40%, the particle loss rate increases abruptly with increasing confining stress. The inflection point corresponds to a confining stress of 2.55 MPa, with a particle loss rate of 15%. When the seepage velocity and confining stress are below the inflection points, particle loss is not affected by these two factors. Conversely, as seepage velocity and confining stress increase, the particle loss rate rapidly increases in a parabolic manner, leading to greater particle loss. When the seepage velocity exceeds 3.0 m/s and the confining stress exceeds 8 MPa, the influence of seepage velocity and confining stress on particle loss gradually diminishes.

### 7.3. Group C gradation

When the content of Group C gradation is less than 40%, the particle loss rate gradually increases with increasing confining stress. The sudden change point in particle loss rate corresponds to a confining stress of 2.3 MPa, with a particle loss rate of 22%. The particle loss rate exhibits sudden increase with increasing seepage velocity. The sudden change point corresponds to a seepage velocity of 2.0 m/s, with a particle loss rate of 14%. When the content of Group C gradation exceeds 40%, the particle loss rate increases abruptly with both increasing confining stress and seepage velocity. The abrupt change point corresponds to a confining stress of 4.45 MPa, with a particle loss rate of 8%, and to a seepage velocity of 3.0 m/s, with a particle loss rate of 45%. When the seepage velocity and confining stress are below the sudden change points, particle loss is not affected by these two factors. Conversely, as seepage velocity and confining stress increase, the particle loss rate increases linearly and rapidly, resulting in greater particle loss. When the confining stress exceeds 8 MPa, its influence on particle loss gradually diminishes.

### 7.4. Warning range

Based on the analysis of particle loss during the evolution of water inrush catastrophe in seepage erosion-induced karst cavities, the warning range for the sudden change in particle loss rate is as follows:

(1) When the content of fine particles such as cuttings and fine sand is less than 60%, the sudden change point in particle loss rate corresponds to a seepage velocity of 2.0 m/s and a confining stress of 4.02 MPa, with a warning range for particle loss of 13%−24%. When the content of fine particles exceeds 60%, the sudden change point corresponds to a seepage velocity of 1.6 m/s and a confining stress of 2.6 MPa, with a warning range for particle loss of 8%−15%. The particle loss rate gradually increases with increasing confining stress and exhibits a sudden increase with increasing seepage velocity.
(2) When the content of coarse particles such as coarse sand and gravel is less than 40%, the abrupt change point in particle loss rate corresponds to a seepage velocity of 2.0 m/s and a confining stress of 2.3 MPa, with a warning range for particle loss of 14%−22%. When the content of coarse particles exceeds 40%, the sudden change point corresponds to a seepage velocity of 3.0 m/s and a confining stress of 4.45 MPa, with a warning value for particle loss of around 45%. The particle loss rate exhibits a sudden increase with both increasing confining stress and seepage velocity.

## 8. Conclusions

This study investigated seepage-erosion-induced water inrush in karst cavities using the SPH method. The effects of particle-size gradation, seepage velocity, and confining stress on the disaster-evolution mechanism were system-atically analyzed, and particle-loss-based early-warning indicators were proposed.

(1) As seepage velocity increases, the overall particle loss rate shows an upward trend. When the flow rate is 1.0 m/s, the total particle loss rate does not exceed 20%, and the fill material is stable, making water inrush unlikely. When the flow rate increases from 1.0 m/s to 2.5 m/s, the total particle loss rate increases by about 70%, and the loss of fill material shifts from gradual to sudden growth. The sudden change point in particle loss rate occurs earlier, but seepage velocity does not affect the abrupt change point in fill material loss, with the particle loss rate remaining around 20%.
(2) Applying confining stress of 2 MPa and 3 MPa to the cavity wall has a minor impact on overall particle loss, with a change in particle loss rate of less than 3% and a sudden change range in particle loss rate of around 25%. When the confining stress increases to above 6 MPa, the particle loss rate rapidly increases by about 20% in the early stage, and the sudden change in fill material loss develops quickly. When the fill material contains a high proportion of coarse particles, the confining stress has a significant impact on water inrush, accelerating the loss of coarse particles in the fill material at the same time, with a maximum loss rate reaching 0.36.
(3) The sudden change in particle loss rate can be considered as the occurrence of water inrush in the cavity. By fitting the particle gradation-confining stress stress-seepage velocity-particle loss rate diagram at the moment of the sudden change point, it is found that the loss of fill material particles is positively correlated with seepage velocity and confining stress. When the content of fine particles such as cuttings and fine sand exceeds 60%, the particle loss rate gradually increases with increasing confining stress and exhibits a sudden increase with increasing seepage velocity. The sudden change point corresponds to a seepage velocity of 1.6 m/s and a confining stress of 2.6 MPa, with a warning range for particle loss of 8%−15%. When the content of coarse particles such as coarse sand and gravel exceeds 40%, the particle loss rate exhibits a sudden increase with both increasing confining stress and seepage velocity. The sudden change point corresponds to a seepage velocity of 3.0 m/s and a confining stress of 4.5 MPa, with a warning value for particle loss of around 45%.

## Supporting information

S1 Table Confining compressive stress and seepage velocity corresponding to sudden change of particle loss rate. (DOCX)
