# Supplementary material for: Catastrophe mechanism and early warning indicators of seepage erosion-induced water inrush in karst cavities
Source: PLoS One. 2026 Jul 31;21(7):e0354695. doi: 10.1371/journal.pone.0354695 (PMC13426919; doi:10.1371/journal.pone.0354695)
Supplement: S1 Table — (DOCX) [file pone.0354695.s001.docx]

**Table S1.** Confining compressive stress and seepage velocity corresponding to sudden change of particle loss rate under particle gradation <0.075 mm（A）

| Particle gradation | | | | | Confining Stress /MPa | Seepage Velocity /(m/s) |
| --- | --- | --- | --- | --- | --- | --- |
| 2-5mm | 0.5-2mm | 0.25-0.5mm | 0.075-0.25mm | <0.075mm |  |  |
| 20% | 20% | 20% | 20% | 20% | 2.30 | 1.99 |
| 15% | 15% | 15% | 15% | 40% | 2.43 | 2.03 |
| 10% | 10% | 10% | 10% | 60% | 2.37 | 1.60 |
| 5% | 5% | 5% | 5% | 80% | 2.60 | 1.63 |

**Table S2.** Confining compressive stress and seepage velocity corresponding to sudden change of particle loss rate under particle gradation 0.075-0.25mm (B)

| Particle gradation | | | | | Confining Stress /MPa | Seepage Velocity /(m/s) |
| --- | --- | --- | --- | --- | --- | --- |
| 2-5mm | 0.5-2mm | 0.25-0.5mm | 0.075-0.25mm | <0.075mm |  |  |
| 20% | 20% | 20% | 20% | 20% | 2.30 | 1.99 |
| 15% | 15% | 15% | 40% | 15% | 4.02 | 2.00 |
| 10% | 10% | 10% | 60% | 10% | 2.43 | 2.06 |
| 5% | 5% | 5% | 80% | 5% | 2.55 | 2.11 |

**Table S3.** Confining compressive stress and seepage velocity corresponding to sudden change of particle loss rate under particle gradation 0.25-0.5mm (C)

| Particle gradation | | | | | Confining Stress /MPa | Seepage Velocity /(m/s) |
| --- | --- | --- | --- | --- | --- | --- |
| 2-5mm | 0.5-2mm | 0.25-0.5mm | 0.075-0.25mm | <0.075mm |  |  |
| 20% | 20% | 20% | 20% | 20% | 2.30 | 1.99 |
| 15% | 15% | 40% | 15% | 15% | 2.00 | 3.00 |
| 10% | 10% | 60% | 10% | 10% | 4.45 | 3.00 |
| 5% | 5% | 80% | 5% | 5% | 4.32 | 3.00 |
